# Multikingdom microbiome-based machine learning enables multiple sclerosis diagnosis

**DOI:** 10.1038/s41522-026-01030-7

**Published:** 2026-05-30

**Authors:** Gaochen Zhu, Guan Yang

**Affiliations:** 1 Department of Infectious Diseases and Public Health, City University of Hong Kong, Hong Kong SAR, China; 2 Shenzhen Research Institute, City University of Hong Kong, Shenzhen, China

**Keywords:** Biomarkers, Computational biology and bioinformatics, Diseases, Microbiology, Neuroscience

## Abstract

Emerging evidence suggests a role for the gut bacteria in the pathogenesis of multiple sclerosis (MS); however, the role of other microorganisms and their diagnostic potential for MS remain poorly explored. Here, we analyzed large-scale metagenomic data derived from fecal samples (discovery cohort *n* = 1152; total *n* = 1306 across 3 geographically diverse cohorts). Subsequently, we utilized multikingdom gut microbiome data to develop machine learning models to distinguish MS patients from healthy controls. Our analysis identified distinct microbiome alterations, revealing 90 bacterial, 3 fungal, 2 viral species, 119 KEGG orthology genes, and 17 metabolic pathways significantly associated with MS. Machine learning models integrating multikingdom taxonomic and functional features achieved the area under the receiver operating characteristic curves (AUCs) of 0.977 for males and 0.978 for females. On external validation datasets, the ensemble models yielded AUCs of 0.813 in males and 0.745 in females, while the 30-marker models reached AUCs of 0.849 and 0.763, respectively. Notably, the accuracy of the model was associated with *Faecalibacterium spp*. and L-methionine biosynthesis pathways, which were less abundant in MS patients. Collectively, our findings highlight the potential application of multikingdom and functional gut microbiome markers as non-invasive biomarkers for MS.

## Introduction

Multiple sclerosis (MS) is a chronic inflammatory and demyelinating disease of the central nervous system that affects over 2.8 million individuals worldwide^1^. Although the exact etiology and pathogenesis of MS remain unknown, it is believed to result from a combination of genetic and environmental factors^2^. Previous research has identified over 200 risk loci associated with MS^3^, with HLA-DRB1*15:01 recognized as the strongest genetic risk factor^3,4^. Interestingly, twin studies comparing MS patients with their unaffected monozygotic twins have revealed a significant role of environmental factors in MS susceptibility^5–7^. Among these factors, gut microbiome dysbiosis has emerged as a potential influence on the severity of MS^8–12^, and experimental autoimmune encephalomyelitis (EAE)^13^, an animal model of MS. Notably, transplantation of fecal microbiome from healthy donors to patients with MS has been shown to improve gastrointestinal motility, neurological function, and urinary function in these patients^14,15^. Conversely, transplantation of microbial contents from MS patients to mice has been associated with the exacerbation of symptoms in EAE^8,9^. These findings suggest a potential correlation between gut microbiome and the development of MS and EAE.

However, these studies primarily concentrated on the alterations in gut bacteria in the pathogenesis of MS, revealing several direct and indirect mechanisms through which bacteria interact with the brain^8,16–18^. It is important to note that other microbial communities, including archaea, fungi, and viruses, also colonize the gut and may play an underestimated role in the disease development^19–21^. While 16S rRNA sequencing is commonly used to identify alterations in bacterial genera among MS patients, this method has limited resolution, which hinders species-specific identification and overlooks non-bacterial microbial kingdoms. In contrast, whole-metagenome shotgun sequencing enables species-level taxonomic profiling and functional characterization across all microbial kingdoms^22–26^.

In this study, we analyzed the multikingdom gut microbiome using metagenomic sequencing data from patients with MS and their paired household healthy controls through the international Multiple Sclerosis Microbiome Study (iMSMS)^27^. We identified key multikingdom microbial markers associated with the onset of MS, developed MS diagnostic models by integrating these microbial markers and functional profiles, and validated the performance of our models using 2 external public datasets^12,28–30^.

## Results

### MS-associated microbial species across four kingdoms and microbiome functions

**Fig. 1:**
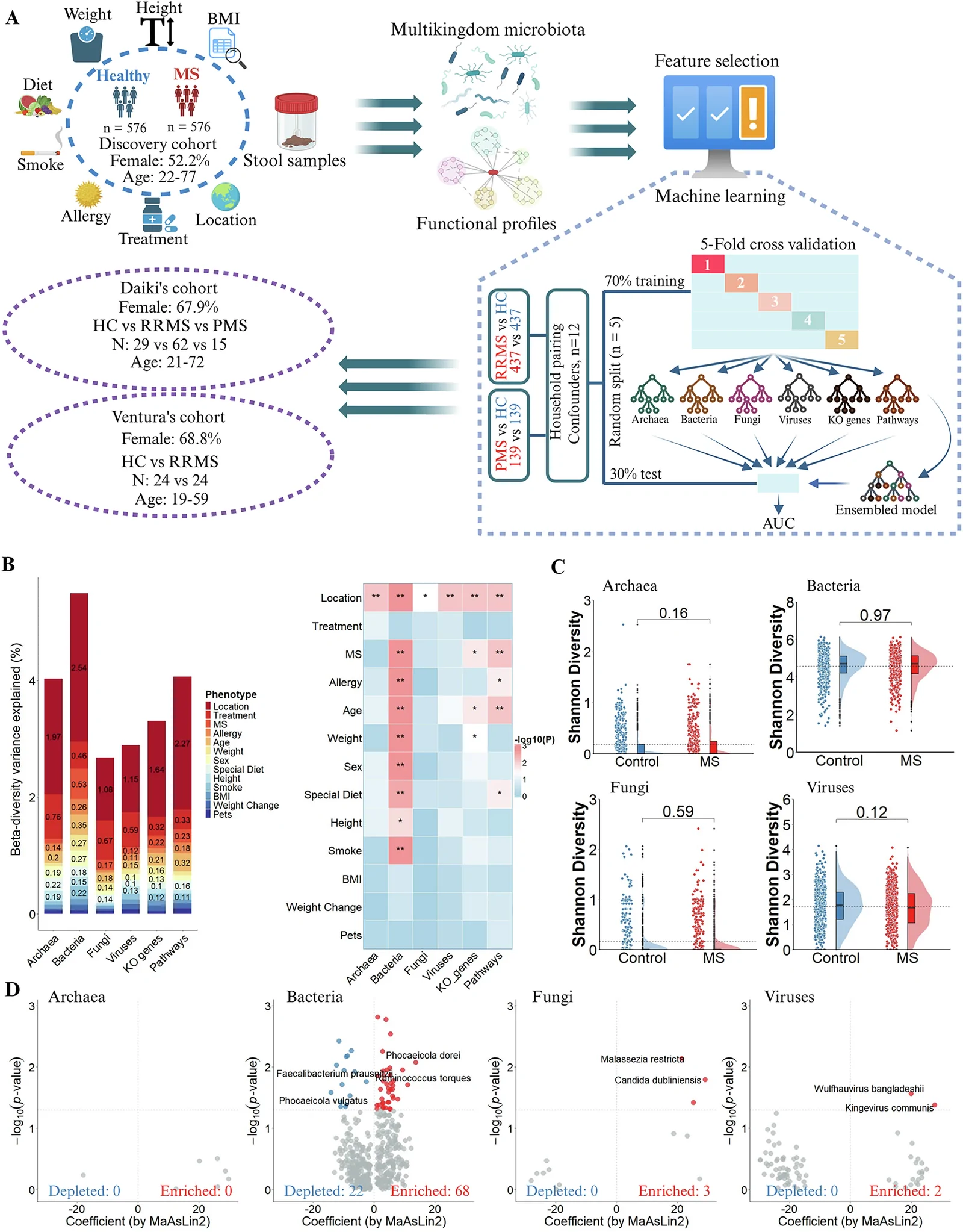
Multikingdom microbiome analysis in patients with MS and paired household healthy controls. **A** Overview of the study workflow. The metagenomic data of the discovery cohort were collected through the International MS Microbiome Study (iMSMS)^27^. **B** PERMANOVA analysis of variance in multikingdom microbiome composition including archaea, bacteria, fungi, and viruses along with associated functions (microbial pathways and genes) for MS. **C** The Shannon index of alpha diversity for the multikingdom microbiome in patients with MS (red, *n* = 576) versus paired household healthy controls (blue, *n* = 576), assessed by FDR-corrected Wilcoxon rank-sum test. **D** Volcano plots depicting associations between multikingdom microbiome species and MS status, as determined by MaAsLin2 analysis, with adjustments for significant confounders. Species with *p* < 0.05 are highlighted in red (enriched in MS) or blue (depleted in MS), with top-ranked species labeled. *p < 0.05, **p < 0.01. Created in BioRender. Gaochen, Z. (2026) https://BioRender.com/tmy12sr.

As gut microbiome composition is linked to environmental and host factors, we first focused on the impact of these factors on the gut microbiome composition to determine potential confounders in each cohort (Fig. 1A). In the discovery cohort, we identified 9 host factors that significantly associated with gut microbial composition, including MS status, sex, age, weight, height, allergy status, special diet needs, location, and smoking status (Fig. 1B, Table S1). Additionally, since diet is a crucial modifier of the gut microbiome^31^, we incorporated all available dietary components into our analysis as covariates. Total caloric intake was represented by the variable “calories”. Among these, 6 dietary components demonstrated significant associations with gut microbial composition, including beta-carotene, carbohydrate, dietary fiber, fat, potassium, and calcium (Fig. S1). In Daiki’s and Ventura’s cohorts, 3 factors-StudyID, MS status, and ethnicity-were associated with microbiome composition (Fig. S2). These variables were treated as confounders and adjusted for in the MaAsLin2 association analysis. For external validation, we further eliminated study-driven batch effects using MMUPHin, with StudyID as the batch variable and MS status as a protected covariate, thereby harmonizing across cohorts while preserving disease-associated signal. In addition, given the known impact of MS treatment on the gut microbiome^32^, treatment status was included as a covariate in downstream analyses when available. Among the patients with MS, 63.7% had received treatment (Table S2).

No differences in Shannon diversity (Fig. 1C) or richness (Fig. S3) were observed across the four kingdoms (archaea, bacteria, fungi, viruses) between MS patients and paired household healthy controls. Using MaAsLin2 with confounder adjustments, we identified 90 bacterial, 3 fungal, and 2 viral species associated with MS (Fig. 1D, Table S3). Notably, among the fungal changes in MS, we observed an enrichment of *Malassezia restricta*, which has been linked to central nervous system impairment in various studies and showed a higher abundance in neurological disorders^33,34^. In the bacterial kingdom, we noted depletion of *Faecalibacterium spp*. and *Blautia spp*., echoing findings from multiple independent cohorts and prior iMSMS observations^10,27,35–37^.

We also assessed the influence of factors on microbiome functions to identify potential confounders across cohorts (Fig. 1B, Fig. S2). In the discovery cohort, 6 host factors was associated with microbiome functions, including MS status, age, weight, allergy status, special diet needs, and location (Fig. 1B). In Daiki’s and Ventura’s cohorts, 2 factors-StudyID and MS status-were associated with changes of microbiome functions (Fig. S2). Using MaAsLin2 with confounder adjustments, we identified 119 KEGG Orthology (KO) gene families and 17 metabolic pathways that were linked to MS (Table S3). Notably, L-methionine-related pathways (e.g., PWY-7977, and PWY-6151) were depleted, consistent with reports linking disrupted methionine metabolism to immune dysregulation and MS pathophysiology^38^. Overall, the gut microbiome of MS patients exhibits significant taxonomic alterations across multiple kingdoms after adjusting for relevant environmental and host confounders, laying the foundation for subsequent development of multikingdom microbiome diagnostic models for MS.

### Single kingdom and multikingdom microbial markers for male-specific MS diagnosis

Bacterial markers for MS diagnosis have been explored in several studies^39,40^. However, the diagnostic potential of archaea, fungi, viruses, microbial genes or function pathways have not been explored in MS. Given that sex is a major determinant in MS microbiome research^41,42^, and was also identified here as a significant factor associated with microbiome variation (Fig. 1B), we next constructed sex-stratified diagnostic models.

**Fig. 2:**
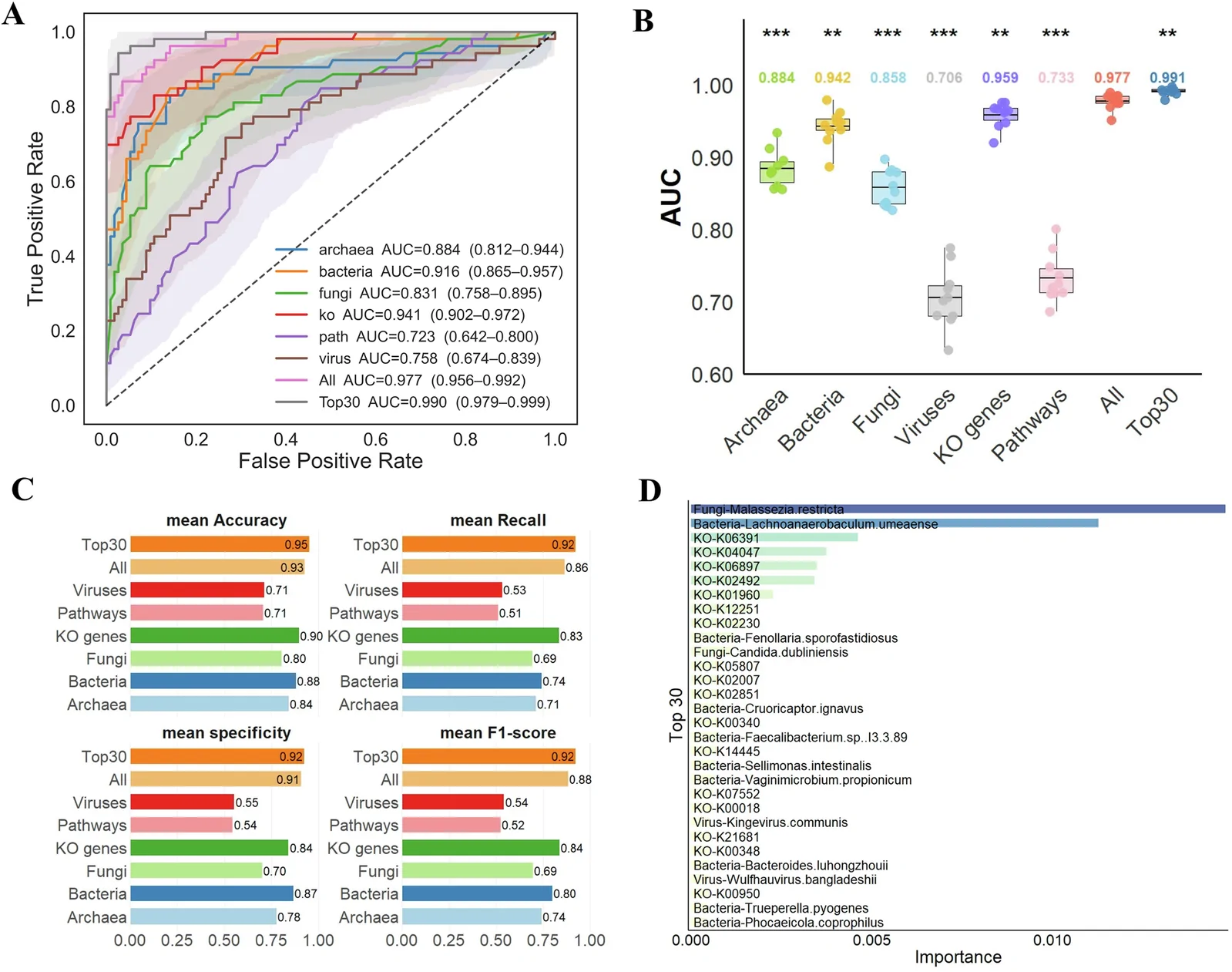
Male-specific random forest models for diagnosing MS. **A** ROC curves of random forest models using different microbial features within the test dataset. **B** Box-and-whisker plot showing the distribution of AUC values obtained from 10 random repetitions of model training and testing. FDR-corrected wilcox rank-sum test was performed between the ensemble model and all other models. **p < 0.01, ***p < 0.001. **C** Comparative analysis of the test set accuracy, recall, specificity and F1-score across models using different microbial features. **D** Importance scores for the top 30 biomarkers identified in the MS classification model trained on all microbial features.

In the discovery cohort, we first evaluated AUCs of models using single-kingdom markers to distinguish male patients with MS from their paired household healthy controls. Among all single-kingdom models, the KO genes model demonstrated the highest predictive capability for detecting MS, achieving an AUC of 0.941 (Fig. 2A), followed by bacteria (AUC 0.916), archaea (AUC 0.884), fungi (AUC 0.831) and viruses-based (AUC 0.758), microbial pathways (AUC 0.723) models (Fig. 2A). We next assessed the performance of a model combining multikingdom features. We found that the ensemble model showed superior diagnostic performance (AUC 0.977) for MS compared with models based on single-kingdom features (Fig. 2A). In 10 random repetitions, the ensemble model consistently demonstrated the highest predictive ability for detecting MS, with an average AUC of 0.977. This was followed by KO genes (AUC 0.959), bacteria (AUC 0.942), archaea (AUC 0.884), fungi (AUC 0.858), microbial pathways (AUC 0.733), and viruses-based (AUC 0.706) models (Fig. 2B). The ensemble model also yielded the highest average accuracy, recall, specificity, and F1-score across repetitions (Fig. 2C). Moreover, the ensemble model exhibited a larger Cohen’s d effect size and higher McFadden’s pseudo-R² than the single-kingdom models (Fig. S4A). These results suggest that the multikingdom taxonomic and functional microbiome biomarker panel offers enhanced diagnostic performance for detecting MS than single-kingdom panels.

To determine the minimal number of microbiome markers required to achieve the highest AUC, we performed permutation importance analysis (Fig. 2D). A total of 30 microbial features yielded an AUC of 0.99 for the diagnosis of MS (Fig. 2A). Additionally, the top 30-marker model showed the highest accuracy, recall, specificity, F1-score, effect size and R² in 10 random repetitions (Fig. 2C, Fig. S4A). Using MaAsLin2, we identified 4 markers that were depleted whereas 25 markers that were enriched in patients with MS (Fig. S5). Notably, *Faecalibacterium sp. I3-3-89* was significantly depleted across the discovery, and external cohorts, with consistent trends observed. Collectively, our results identify the multikingdom microbiome as a promising biomarker for MS.

### Single kingdom and multikingdom microbial markers for female-specific MS diagnosis

**Fig. 3:**
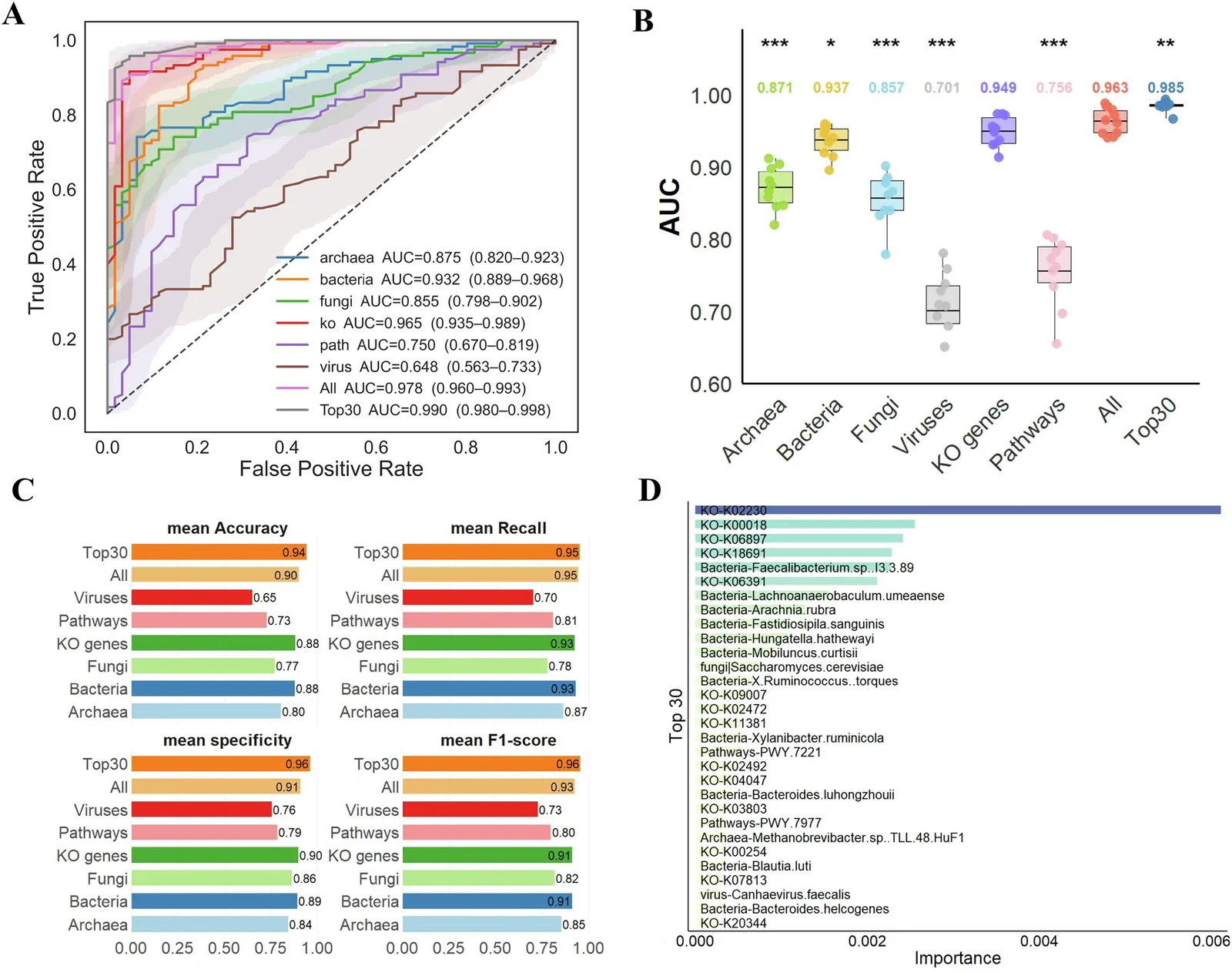
Female-specific random forest models for diagnosing MS. **A** ROC curves of random forest models using different microbial features within the test dataset. **B** Box-and-whisker plot showing the distribution of AUC values obtained from 10 random repetitions of model training and testing. FDR-corrected wilcox rank-sum test was performed between the ensemble model and all other models. *p < 0.05, **p < 0.01, ***p < 0.001. **C** Comparative analysis of the test set accuracy, recall, specificity and F1-score across models using different microbial features. **D** Importance scores for the top 30 biomarkers identified in the MS classification model trained on all microbial features.

In the discovery cohort, we also evaluated the AUCs of models using single-kingdom markers to distinguish female patients with MS from the paired household healthy controls. Among all single-kingdom markers, the KO genes model demonstrated the highest predictive capability for detecting MS, achieving with an AUC of 0.965 (Fig. 3A), followed by bacteria (AUC 0.932), archaea (AUC 0.875), fungi (AUC 0.855), microbial pathways (AUC 0.75), viruses-based (AUC 0.648) models. We next assessed the performance of the model combining multikingdom features. We found that the ensemble model showed superior diagnostic performance (AUC 0.978) for MS compared with models based on single-kingdom features (Fig. 3A). Ten repeated random splits further supported that the ensemble model consistently outperformed single-kingdom models (Figs. 3B, C, S4B). Permutation importance analysis identified a 30-feature panel yielding an AUC of 0.99 (Fig. 3A, D) and the strongest average accuracy, recall, specificity, F1-score, effect size and R² (Figs. 3C, S4B). Based on MaAsLin2 analysis, we identified 9 markers that were depleted and 18 markers that were enriched in patients with MS (Fig. S5). Notably, *Faecalibacterium sp. I3-3-89, Blautia luti*, and PWY-7977 (L-methionine biosynthesis IV) were significantly depleted in the discovery cohort and showed the same trends in the external cohort. The observation that *Faecalibacterium sp. I3-3-89* ranked among the top markers in both male and female models suggests that it may represent a shared microbiome signature of MS across sexes. Taken together, we establish the potential of the multikingdom microbiome-based model as a promising diagnostic tool for MS.

### Validation of multikingdom-based models for MS diagnosis in independent cohorts

**Fig. 4:**
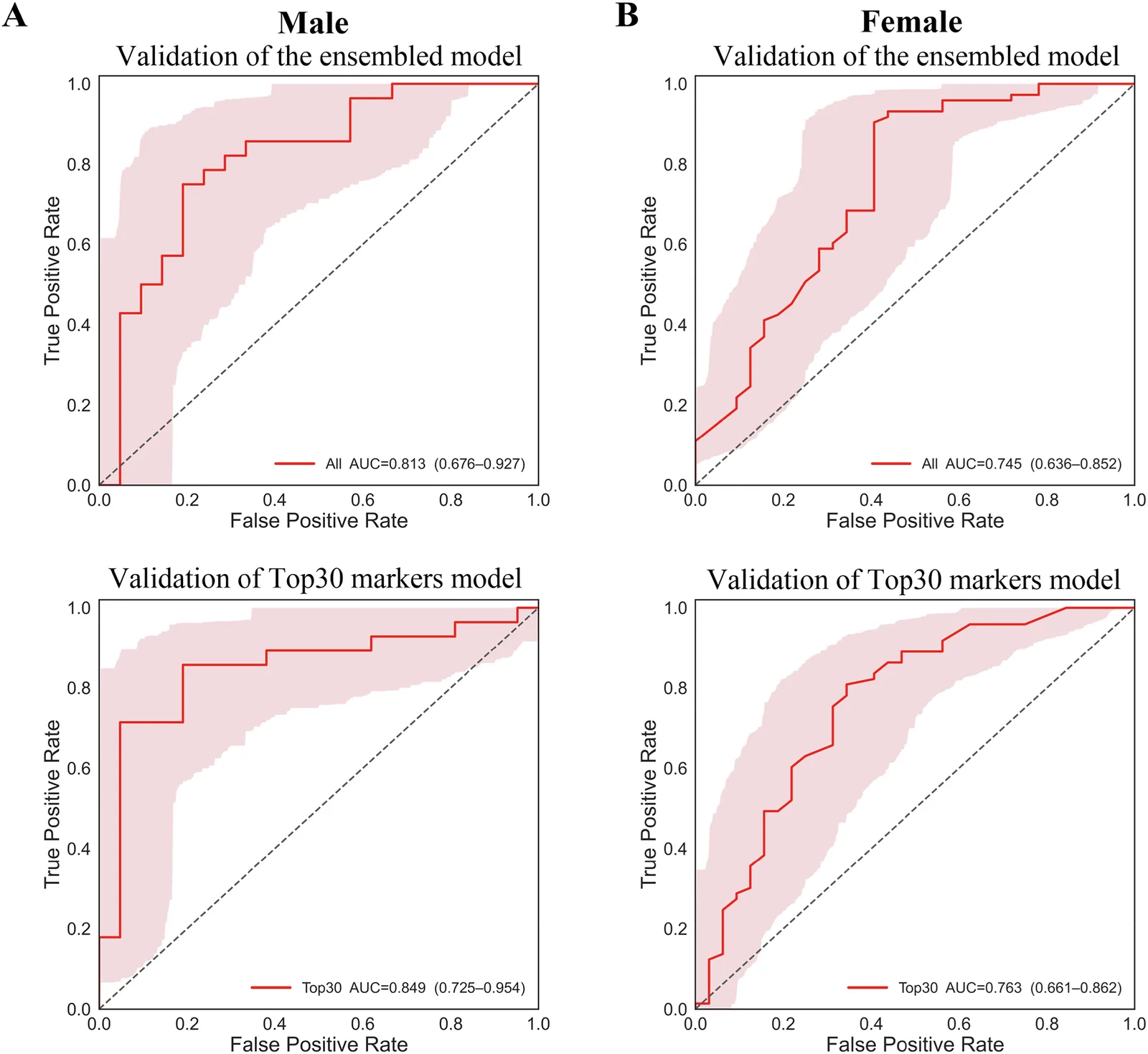
Validation of random models on external validation datasets. ROC curves of male-specific MS diagnostic model using all microbial features and top 30 features (**A**) and female-specific diagnostic model using all microbial features and top 30 features (**B**) on external validation datasets.

To externally validate the diagnostic value and mitigate the risk of over-optimistic reporting of diagnostic accuracy, we assessed the ensemble model and top marker model across 2 independent public datasets after MMUPHin-based batch correction. In the integrated external cohort, the ensemble male-specific MS model showed an AUC of 0.813 and the 30-marker model reached an AUC of 0.849 (Fig. 4A).

For female-specific MS diagnosis, the ensemble model achieved an AUC of 0.745 and the 30-marker model reached an AUC of 0.763 (Fig. 4B) in the external cohort. Overall, these results indicate that the models retained discriminatory ability in independent cohorts.

### Multikingdom-based models for MS subtypes diagnosis

We further evaluated models for MS subtypes (relapsing-remitting multiple sclerosis, RRMS; and progressive multiple sclerosis, PMS) stratified by sex. In RRMS, we identified 10 host factors and 18 dietary components associated with microbial composition (Fig. S6A, B), whereas in PMS, 6 host factors and 6 dietary components were associated with composition (Figure S7A, B). We also evaluated the factors influencing microbiome functions in RRMS and PMS patients (Fig. S6, S7). These covariates were adjusted for in the MaAsLin2 pipeline.

In the discovery cohort, the male RRMS ensemble model achieved an AUC of 0.949 (Fig. S8A), and a 30-marker panel achieved an AUC of 0.979 (Fig. S8A, B). In the external RRMS cohort, performance remained predictive (ensemble AUC = 0.768; 30-marker AUC = 0.801; Fig. S8C, D). For female RRMS, the ensemble model achieved an AUC of 0.958 and the 30-marker panel achieved an AUC of 0.986 (Fig. S9A, B), with external performance of 0.725 (ensemble) and 0.715 (30-marker) (Fig. S9C, D).

For PMS, we performed the same analyses (Fig. S10, S11). The 30-marker models yielded the highest AUCs within discovery cohort (Fig. S10A, B and Fig. S11A, B). In the external PMS cohort, performance was lower than for overall MS and RRMS (male: ensemble AUC = 0.597; 30-marker AUC = 0.677; Fig. S10C, D; female: ensemble AUC = 0.617; 30-marker AUC = 0.652; Fig. S11C, D). We speculate that reduced external performance in PMS reflects limited sample size and heterogeneity in progressive disease, which may reduce statistical power during feature selection and impair model generalization. Nevertheless, these results suggest the feasibility of using multikingdom microbiome markers to classify MS subtypes and highlight the need for larger progressive MS cohorts for further refinement.

## Discussion

Previous studies have predominantly focused on gut bacterial alterations in MS. However, recent investigations have illuminated the critical roles of non-bacterial microorganisms within the gut-brain axis^19–21^. Despite their significance, these microorganisms have been infrequently studied in the context of MS. In this study, we conducted an integrated analysis of the multikingdom and microbiome functions using 1306 metagenomes across 3 independent cohorts. We observed numerous differences in microbial composition across multiple kingdoms and microbiome functions between MS patients and healthy controls. We demonstrated that diagnostic models based on multikingdom markers achieved high predictive values for MS diagnosis, highlighting the multifaceted role of the gut microbiome in MS pathogenesis and its potential for non-invasive diagnostics. Importantly, our findings suggest that microbial functional information is not merely complementary to taxonomy, but may capture disease-relevant significance that is less apparent from taxonomic shifts alone. The consistently strong performance of KO gene-based models, together with the added benefit of multikingdom integration, supports the view that MS-associated dysbiosis is best understood as a combined ecological and functional perturbation.

In addition to alterations of *Hungatella hathewayi*, *Faecalibacterium prausnitzii*, and some other previously reported bacterial species, we also discovered fungal, and viral species associated with MS. However, limited studies have examined the association between these species with MS development^19–21^. Despite the limited sensitivity of shallow shotgun sequencing for detecting non-bacterial features, these findings nevertheless provide an exploratory view of the broader microbial landscape. Further exploration may be required to better understand the functional roles of these species in MS pathogenesis. Among the top-ranking features, the repeated identification of *Faecalibacterium spp*., including *Faecalibacterium sp. I3-3-89*, in both male and female models is particularly noteworthy. This convergence across sex-stratified analyses suggests that depletion of *Faecalibacterium* may represent a shared microbial signature of MS rather than a cohort-specific or sex-specific finding. This interpretation is biologically plausible, as members of the genus *Faecalibacterium*, particularly *F. prausnitzii*, are widely recognized as anti-inflammatory commensals that contribute to intestinal homeostasis through short-chain fatty acid production, maintenance of epithelial barrier integrity, and modulation of host immune responses^43,44^. Reduced abundance of *Faecalibacterium* has been reported in MS and other chronic inflammatory conditions, supporting the possibility that loss of this genus reflects diminished anti-inflammatory buffering within the gut ecosystem^36,43^. Prior mechanistic studies have also shown that gut microbiota from MS patients can modulate T-cell responses and exacerbate neuroinflammation in experimental systems^8,9^, while recent large-scale work from the iMSMS consortium further supports a role for the gut microbiome in peripheral immune activation and microglial pathology in MS^27^. Although our study was not designed to establish a causal role for *Faecalibacterium sp. I3-3-89* specifically, its recurrence across sexes and cohorts supports its candidacy as a promising biomarker and raises the possibility that loss of *Faecalibacterium*-associated anti-inflammatory functions is a relevant component of MS pathophysiology. Moreover, we found that certain metabolic pathways in the top markers panel that may contribute to MS pathogenesis via dysregulation of methionine metabolism^38^. Compared with taxonomy-based markers, pathway-level features may better capture convergent microbial functions across distinct community structures. While these findings require further metabolomic and mechanistic validation, they suggest that functional readouts may provide a biologically informative and potentially more transferable biomarker layer than taxonomy alone.

Although several studies have attempted to identify reproducible microbial biomarkers for MS screening^30,45,46^, adequate validation across different cohorts remains limited. The inconsistency in findings raises critical questions about whether microbial alterations reflect intrinsic biological differences among cohorts, experimental biases, or insufficient statistical power to facilitate meaningful comparisons. To address these concerns, we systematically evaluated the impact of host and environmental variables on gut microbiome using MS metagenomic datasets, fully adjusting for identified confounders throughout our analysis. After adjustment, we demonstrated that models incorporating multikingdom and microbiome functional markers exhibited superior diagnostic accuracy compared to those based solely on single-kingdom markers. This is particularly noteworthy given that the inclusion of microbiome functional data has been shown to provide significant diagnostic and prognostic value in other human diseases^47,48^.

To minimize the impact of environmental and host factors on our diagnostic model, we constructed a household-controlled training cohort comprising MS patients and paired household healthy controls^27^, thereby reducing the potential influence of confounding variables. In addition, we analyzed MS cohorts characterized by diverse lifestyles, ethnicities, and geographic locations, thus achieved a more comprehensive representation of the spectrum of MS cases and controls. We further validated both ensemble and top-marker models in two independent public datasets after MMUPHin-based batch correction. The models retained moderate discriminatory ability across cohorts, supporting their predictive potential while also underscoring the challenges of cross-cohort generalization in microbiome-based diagnostics. The markedly higher performance in the discovery cohort than in the external validation cohorts also suggests possible overfitting and feature instability, and may additionally reflect residual heterogeneity in population characteristics, sample processing, and sequencing across studies.

We acknowledge there are some limitations of our diagnostic model. First of all, although the iMSMS cohort consisted of household-paired samples, this was not available in the external cohorts, which may have affected model performance during external validation. Second, the smaller sample size for PMS (154 patients) compared to RRMS (716 patients) may have restricted the detection of subtype-specific signatures and reduced model robustness. Third, shallow shotgun sequencing may have limited the resolution of low-abundance community members, especially non-bacterial species. Future studies using deeper sequencing and kingdom-targeted protocols may be needed to better define the non-bacterial microbiome in MS. In addition, the discovery cohort was substantially larger than the combined external validation cohort (1152 vs 154 samples), which likely contributed to differences in statistical power. Future research should include larger, more diverse cohorts with comprehensive metadata and incorporate additional host and environmental features to enhance model generalizability and precision.

In conclusion, this study suggests that multikingdom and functional gut microbiome markers can be utilized for non-invasive MS diagnosis, facilitating candidate biomarker panels for future clinical validation.

## Methods

### Data collection and processing

The workflow of the whole study is summarized in Fig. 1A. Human shallow metagenomic sequencing data of the discovery cohort were collected from EMBL-ENA repository with accession number ERP115476 through the International Multiple Sclerosis Microbiome Study^27^. Shallow shotgun sequencing with 0.5 million sequences per sample has been shown to be a powerful alternative to whole metagenome sequencing^22–26^. A total of 1152 participants (aged 18–81 years, 52.2% female) were included in this study. Among these, 576 samples were collected from the participants diagnosed with RRMS or primary progressive MS (PPMS) or secondary progressive MS (SPMS), while 576 non-MS samples were collected from paired household healthy controls (HC) (Tables S2, S4). Given the lack of significant differences across various metrics for 4 microbial kingdoms (Fig. S12), together with the relatively small sample size of SPMS and PPMS compared with RRMS, these two groups were combined into a single category, referred to as PMS for subsequent analyses. Comprehensive phenotypic metadata, including age, sex, weight, height, BMI, allergy status, diet, sampling site, smoking status, weight change, pet ownership, and treatment status were collected (Fig. 1A). Additionally, 154 fecal metagenomes (aged 19–66 years, 68.2% female) from 2 published datasets^12,28–30,49^ were included for external validation (Fig. 1A, Tables S4, S5). To ensure consistency across datasets, we referred to each cohort by the first author’s name: Daiki’s cohort (HC = 29, RRMS = 62, SPMS = 15), and Ventura’s cohort (HC = 24, RRMS = 24). These cohorts represented diverse geographical regions, including the United States, Argentina, the United Kingdom, Spain, and Japan, ensuring a global representation. Notably, females constituted 69.9% (473/677) of MS patients, consistent with the known female predominance in MS epidemiology. We retrieved raw sequencing data through accession numbers provided in the publications and downloaded metadata directly from publications if available. In the external validation analyses, StudyID denotes the source cohort from which each sample was derived (e.g., Daiki’s cohort or Ventura’s cohort).

### Microbial taxonomic and functional profiles

Raw sequencing data were processed using Trimmomatic (v0.39) to remove adapter sequences and low-quality reads (Phred score < 30). To eliminate host genomic contamination, high-quality reads were filtered using KneadData (v0.12.0). Taxonomic classification of bacteria, archaea, fungi, and viruses was performed using Kraken 2 (v2.1.3), a k-mer-based metagenomic classifier optimized for accuracy^50^. To balance sensitivity with classification precision, we applied a Kraken2 confidence threshold of 0.2, consistent with common practice in metagenomic analyses^51^. Species-level abundance estimates were refined using Bracken (v2.5.0) to improve taxonomic resolution^52^. The reference database was the NCBI Kraken 2 Standard Database (January 2024).

To reduce the influence of low-confidence features, we adopted a pre-screening protocol to eliminate taxonomic features whose average read count < 5 or genome coverage breadth < 20%. These thresholds were also employed by other microbiome studies^53,54^.

Functional profiling of microbial pathways and KO gene families was conducted using HUMAnN (v3.7), with results transformed into relative abundances^55^. The microbiome data were transformed via the cumulative sum scaling transformation, a median-like quantile normalization which corrects differences in sampling depth^56^.

### Calculation of microbiome–phenotype associations

The proportion of variance in microbiome composition explained by phenotypic factors was assessed using permutational multivariate analysis of variance (PERMANOVA) based on Bray–Curtis distance matrices, as implemented in the adonis2 function of the R package vegan (v2.6.8) with 9999 permutations^57^. Marginal tests were performed using “by = margin” so that each variable was evaluated while adjusting for the others in the analyses. Beta-diversity was calculated from relative abundances of microbial species to evaluate community-level differences.

Similarly, the variance in microbial functional potential was analyzed using Bray–Curtis distance matrices derived from MetaCyc pathways and KO gene abundances. Phenotypic factors with significant associations (*p* < 0.05) were identified as confounders and adjusted for in subsequent analyses.

Alpha-diversity metrics, including the Shannon index and species richness (observed species count), were computed to assess within-sample diversity. Associations between microbial features (species, MetaCyc pathways, and KO genes) and MS status were evaluated using MaAsLin2 (v1.16.0)^58^, adjusting for confounders identified in PERMANOVA. MaAsLin2 employed general linear models with appropriate normalization and transformation methods to ensure statistical inference. We modeled MS status while jointly controlling for host, dietary, and medication variables, and additionally extracted MaAsLin2 residuals as covariate-adjusted feature matrices to minimize confounding-driven separability in downstream analyses. For cross-cohort validation, batch effects attributable to study were removed using MMUPHin (v1.16.2)^59^ via adjust_batch() with StudyID specified as the batch factor and MS status included as a protected biological covariate, thereby harmonizing feature distributions across studies while preserving disease-associated signal. For PERMANOVA and differential abundance testing in MaAsLin2, multiple-testing correction was performed using the Benjamini-Hochberg false discovery rate procedure. The workflow of data preprocessing was shown in Fig. S13.

### Random forest classifier

A random forest classifier was developed using scikit-learn (v1.2.2) in Python (v3.9) to distinguish MS patients from controls based on microbial features. Random forest was selected due to its superior performance in microbiome data analysis in previous studies^47,60^. Features were derived from taxonomic profiles (bacteria, archaea, fungi, viruses) and functional profiles (KO genes, MetaCyc pathways), with unique prefixes to avoid naming conflicts and z-score standardization. Feature selection was performed before model training using discovery-cohort differential analyses, and only features retained after abundance/prevalence filtering and association-based screening were entered into model construction. For archaeal, fungal, and viral feature sets, the numbers of retained features after pre-screening were relatively small (7, 13, and 82, respectively), and therefore all retained features were used for model training. The dataset was divided into training (70%) and testing (30%) sets, with stratified sampling to maintain class balance. Hyperparameters (number of estimators, maximum depth, maximum features, minimum sample splits, minimum sample leaves, class weights) were optimized using grid search with 5-fold cross-validation to prevent overfitting. To address class imbalance, Synthetic Minority Over-sampling Technique (SMOTE) was applied during model training^61^. Household pairs were kept grouped in the 70/30 split and 5-fold cross-validation. Model performance was evaluated on the test set using AUC. Individual models were trained for each feature type, with ROC curves comparing discriminative abilities. With the best combination of hyperparameters, the random split of the cohort was repeated 10 times to obtain a distribution of random forest prediction evaluations on the test set. Feature importance was assessed using SHAP or permutation analysis to identify key biomarkers, thereby enhancing interpretability^62,63^. Method that achieved higher model performance was used. If a training feature was absent in an external validation dataset, its value was set to zero during prediction.

### Statistical analysis

All statistical analyses were conducted using R software (v.4.3.1). For each microbial kingdom, the Shannon diversity and richness between the healthy control and MS groups were compared using the Mann–Whitney U test with FDR correction for multiple testing, a non-parametric method suitable for comparing two independent groups without assuming normal distribution. Statistical significance was set at three levels: *p < 0.05, **p < 0.01, and ***p < 0.001.

### Ethical approval

The study was approved by the Human Subject Ethics Sub-Committee of the Jockey Club College of Veterinary Medicine and Life Sciences (jcc2425ay001), City University of Hong Kong.

## Supplementary information

Supplementary Materials_v2.

Table S1 & Table S2 & Table S5.

Table S3.

Table S4

## Data Availability

All study data are included in the article and/or SI Appendix. The metagenomes used in this work can be accessed at NCBI (accessions: PRJEB28543) and EMBL-ENA (accessions: ERP115476, DRA007704, DRA017647).

## Code availability

All software used is from publicly available sources. Codes used for the microbiome analyses or figures are available via GitHub at https://github.com/zgc-ml/Multikingdom-microbiome-based-machine-learning-for-multiple-sclerosis-diagnosis.
