# Supplementary material for: Multikingdom microbiome-based machine learning enables multiple sclerosis diagnosis
Source: NPJ Biofilms Microbiomes. 2026 May 30;12:166. doi: 10.1038/s41522-026-01030-7 (PMC13519030; doi:10.1038/s41522-026-01030-7)
Supplement: Supplementary file 1 [file 41522_2026_1030_MOESM1_ESM.pdf]

Supplementary Figures for

Title: Multikingdom microbiome-based machine learning enables multiple sclerosis diagnosis

Authors: Gaochen Zhu[1], Guan Yang[1,2,]*

Affiliation: [1]Department of Infectious Diseases and Public Health, City University of Hong Kong, Kowloon, Hong Kong SAR 999077, China. [2]Shenzhen Research Institute, City University of Hong Kong, Shenzhen 518057, China.

***Corresponding Author:** Guan Yang, gyang25@cityu.edu.hk

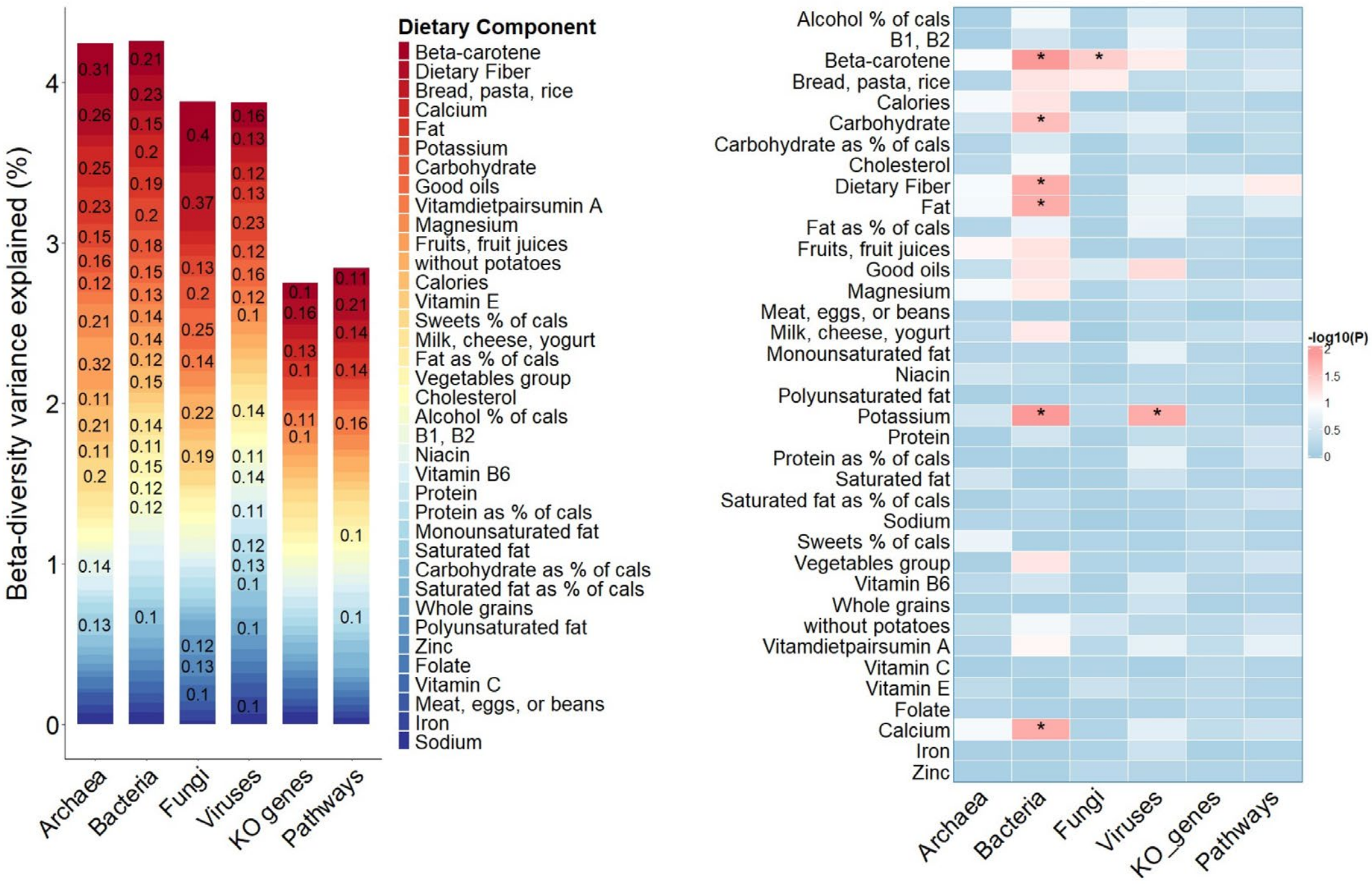

Figure S1. Variance in multikingdom microbiome composition and functions explained by dietary components in the discovery cohort. PERMANOVA analysis of variance explained by 37 dietary components in multikingdom microbiome composition including archaea, bacteria, fungi, and viruses along with associated functions (microbial pathways and genes) for MS. *$p < 0.05$.

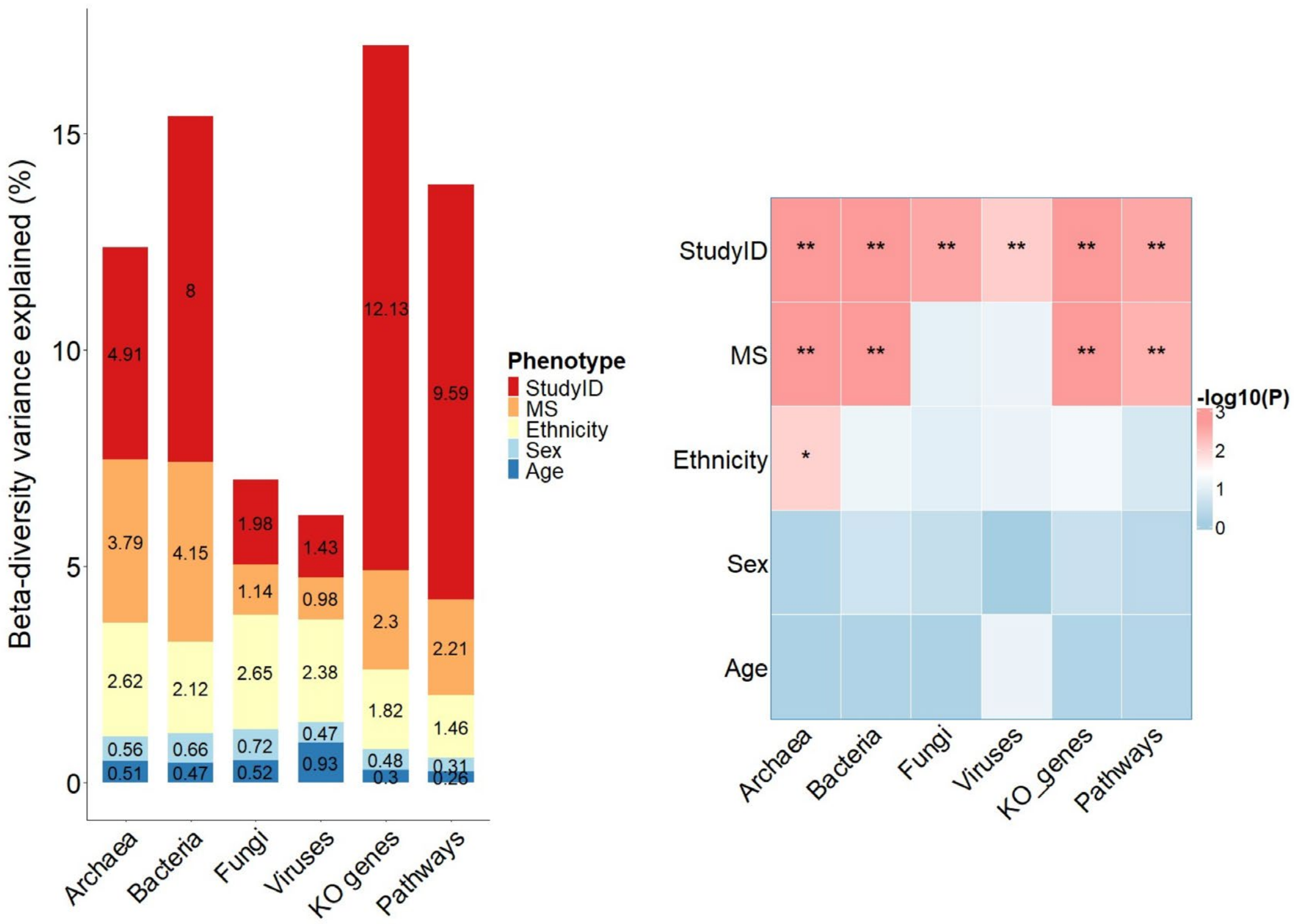

Figure S2. Variance in multikingdom microbiome composition and functions explained by host factors in external MS cohorts. PERMANOVA analysis of variance in multikingdom microbiome composition including archaea, bacteria, fungi, and viruses along with associated functions (microbial pathways and genes) for MS in Ventura's and Daiki's cohorts. *$p < 0.05$, **$p < 0.01$.

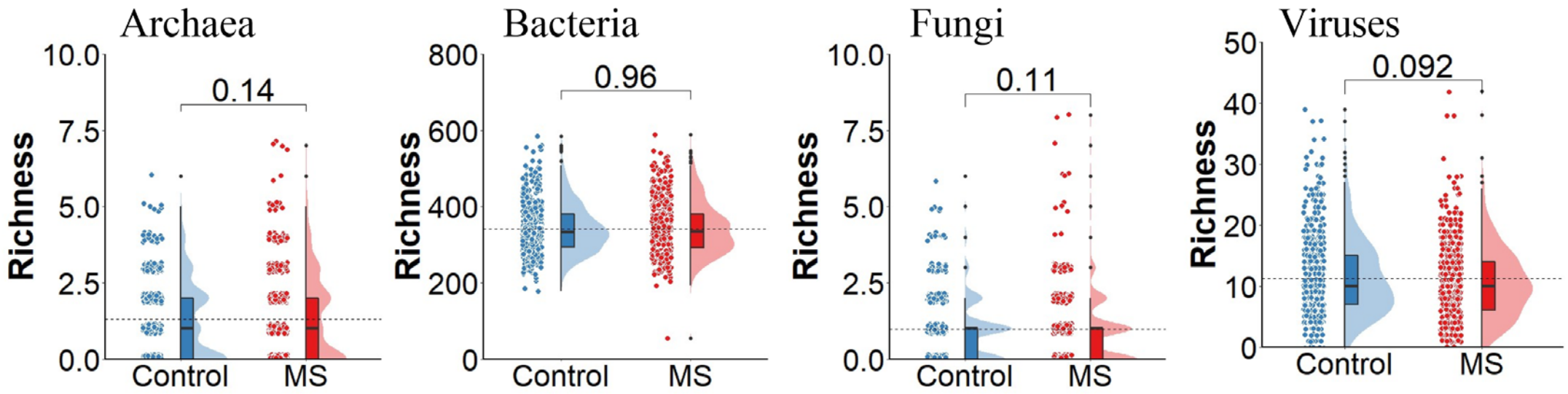

Figure S3. The richness of alpha diversity for the multikingdom microbiome in patients with MS (red, n = 576) versus paired household healthy controls (blue, n = 576), assessed by FDR-corrected Wilcoxon rank-sum test.

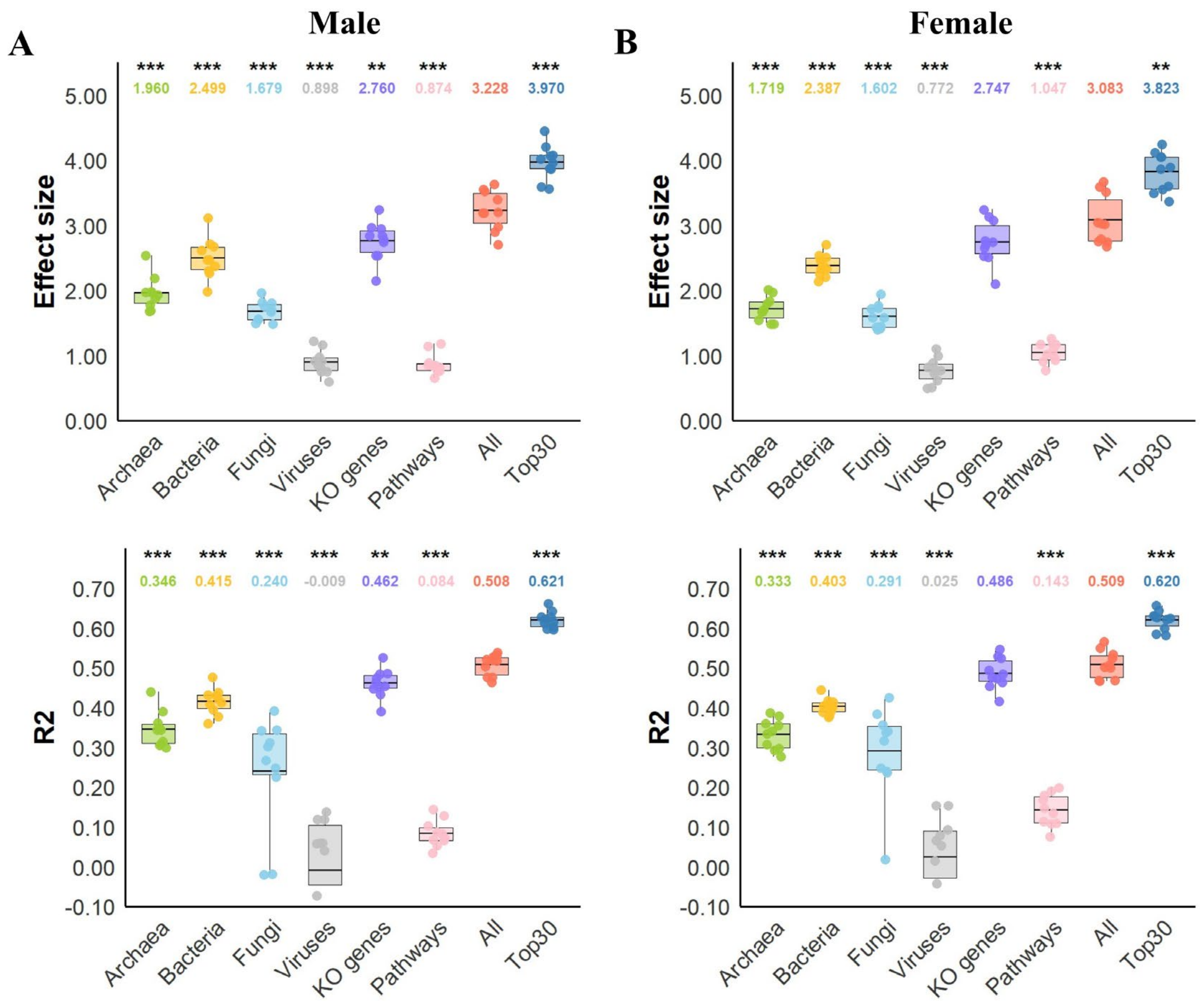

Figure S4. Box-and-whisker plot showing the distribution of Cohen's d effect size and McFadden's pseudo-R^2 values obtained from 10 random repetitions of model training and testing for (A) male and (B) female. FDR-corrected wilcox rank-sum test was performed between the ensemble model and all other models. $^{**}p < 0.01$, $^{***}p < 0.001$.

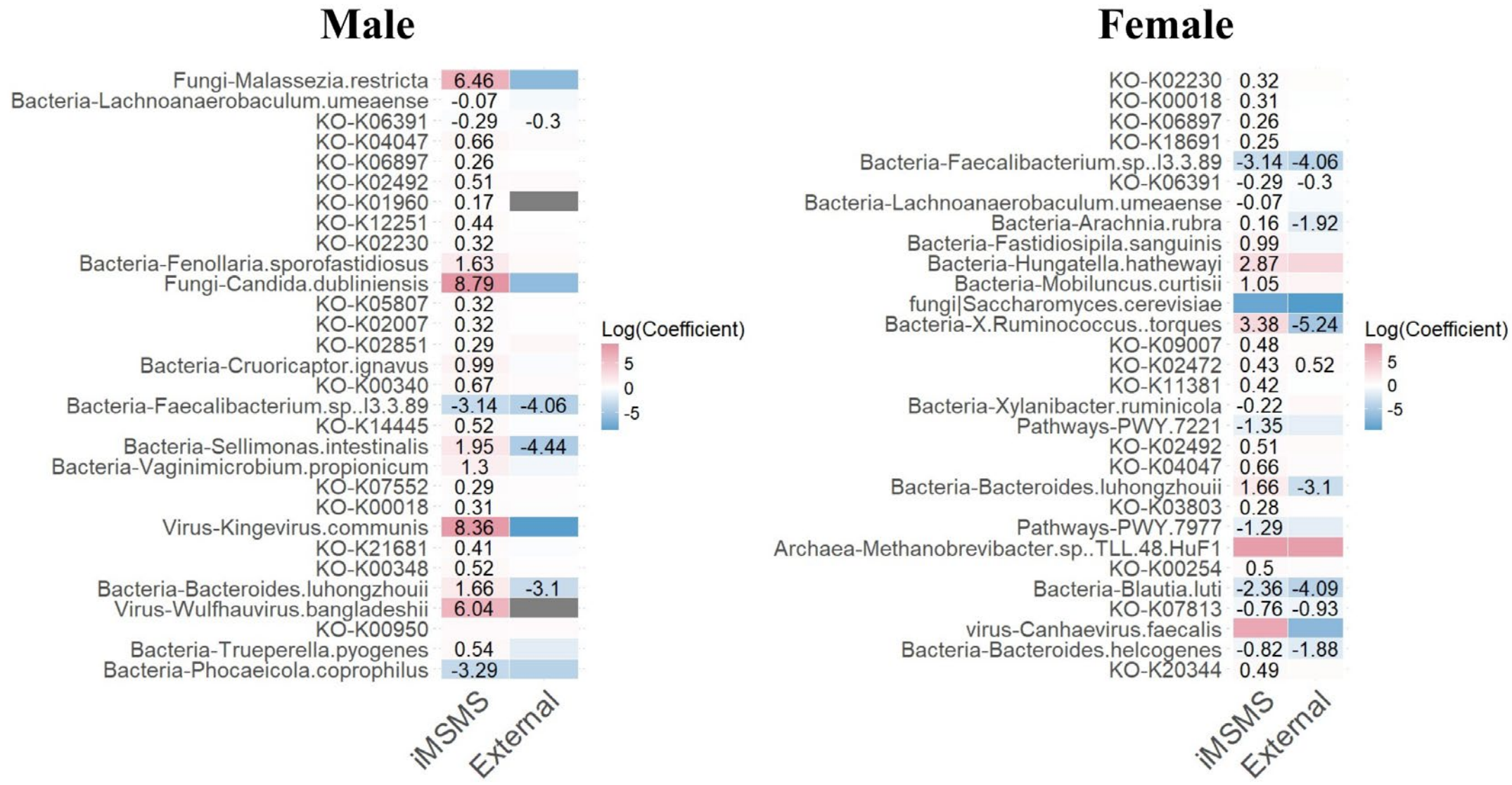

Figure S5. Associations between MS and the top 30 fecal microbiome biomarkers across iMSMS and external cohorts, as determined by MaAsLin2 analysis; coefficients are presented for associations with a $p < 0.05$.

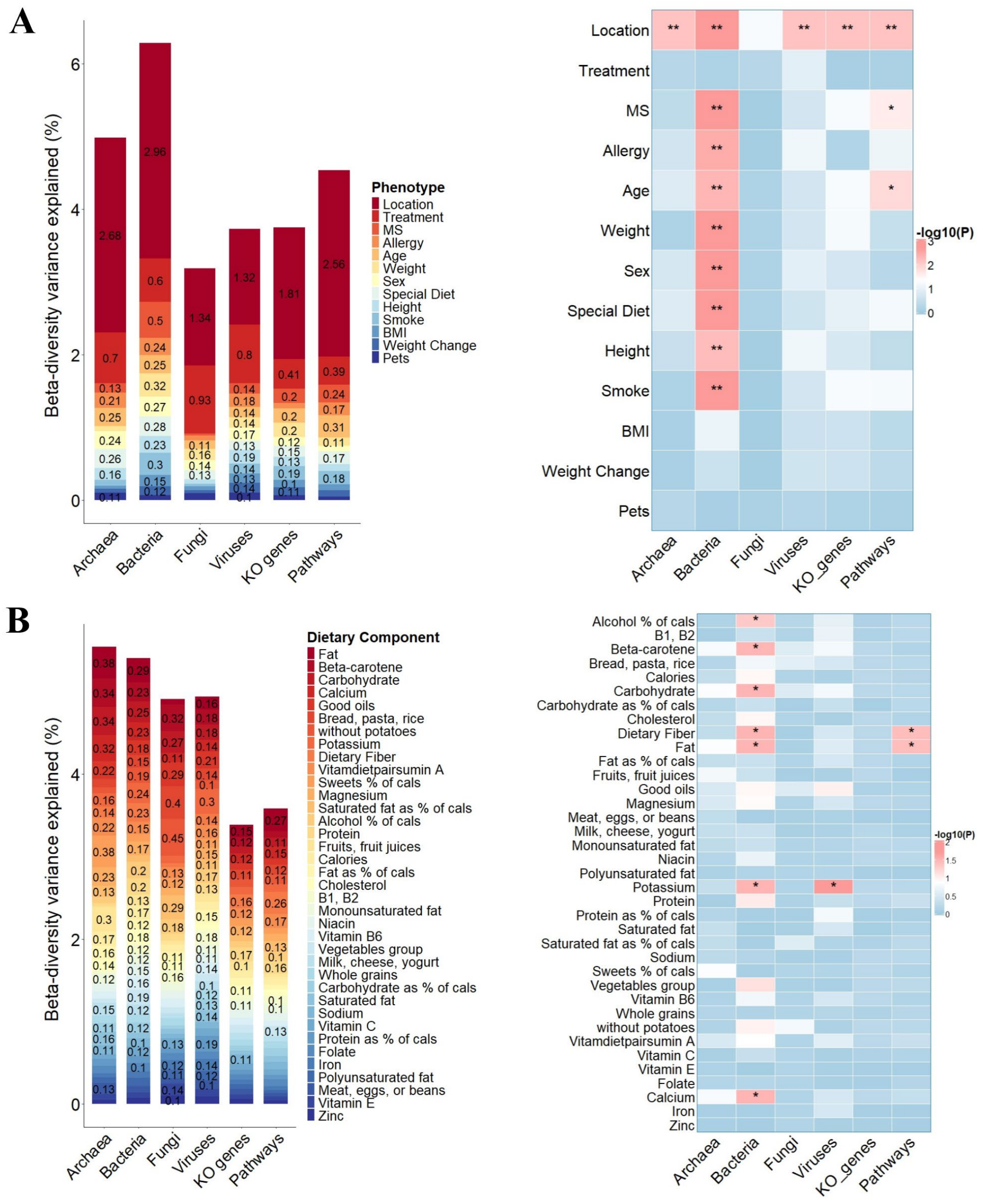

Figure S6. Variance in multikingdom microbiome composition and functions explained by host factors and dietary components for RRMS in the discovery cohort. PERMANOVA analysis of variance explained by 13 host factors (A) and 37 dietary components (B) in multikingdom microbiome composition including archaea, bacteria, fungi, and viruses along with associated functions (microbial pathways and genes) for RRMS. *$p < 0.05$, **$p < 0.01$.

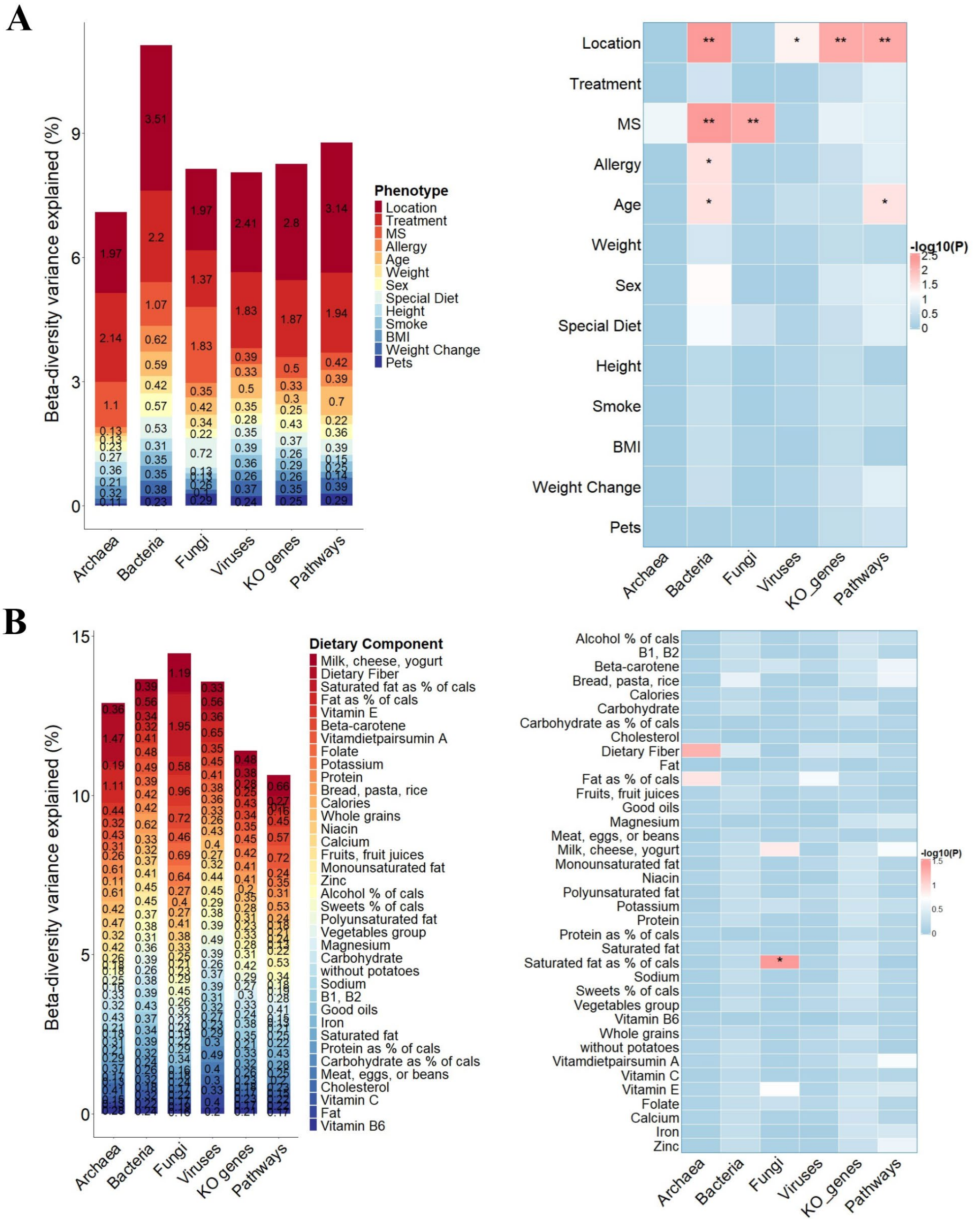

Figure S7. Variance in multikingdom microbiome composition and functions explained by host factors and dietary components for PMS in the discovery cohort. PERMANOVA analysis of variance explained by 13 host factors (A) and 37 dietary components (B) in multikingdom microbiome composition including archaea, bacteria, fungi, and viruses along with associated functions (microbial pathways and genes) for PMS. *$p < 0.05$, **$p < 0.01$.

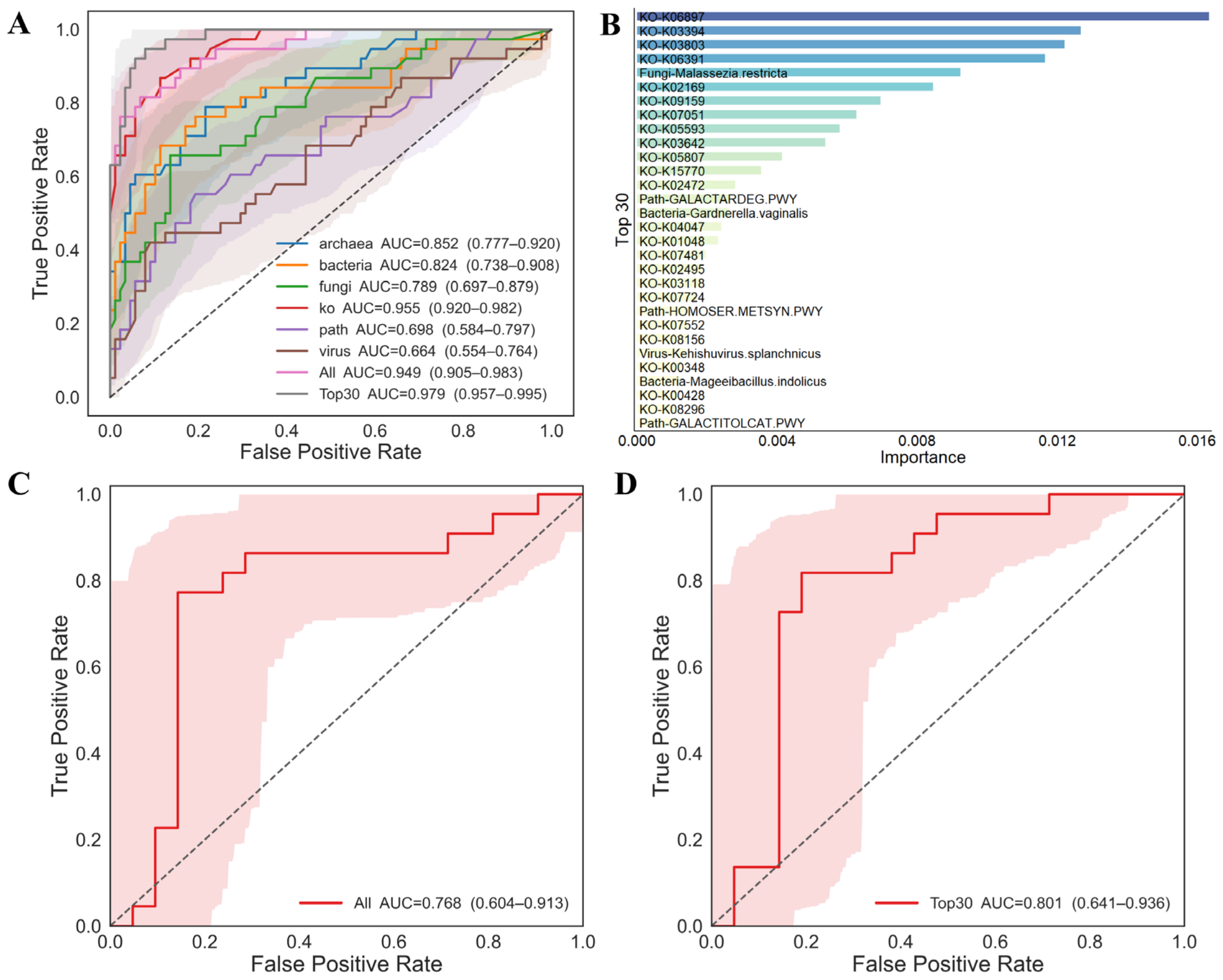

Figure S8. Male-specific random forest models for diagnosing RRMS. (A) ROC curves of random forest models using different microbial features within the test dataset. (B) Importance scores for the top 30 biomarkers identified in the RRMS classification model trained on all microbial features. ROC curves of male-specific RRMS diagnostic model using all microbial features (C) and top 30 features (D) on external validation datasets.

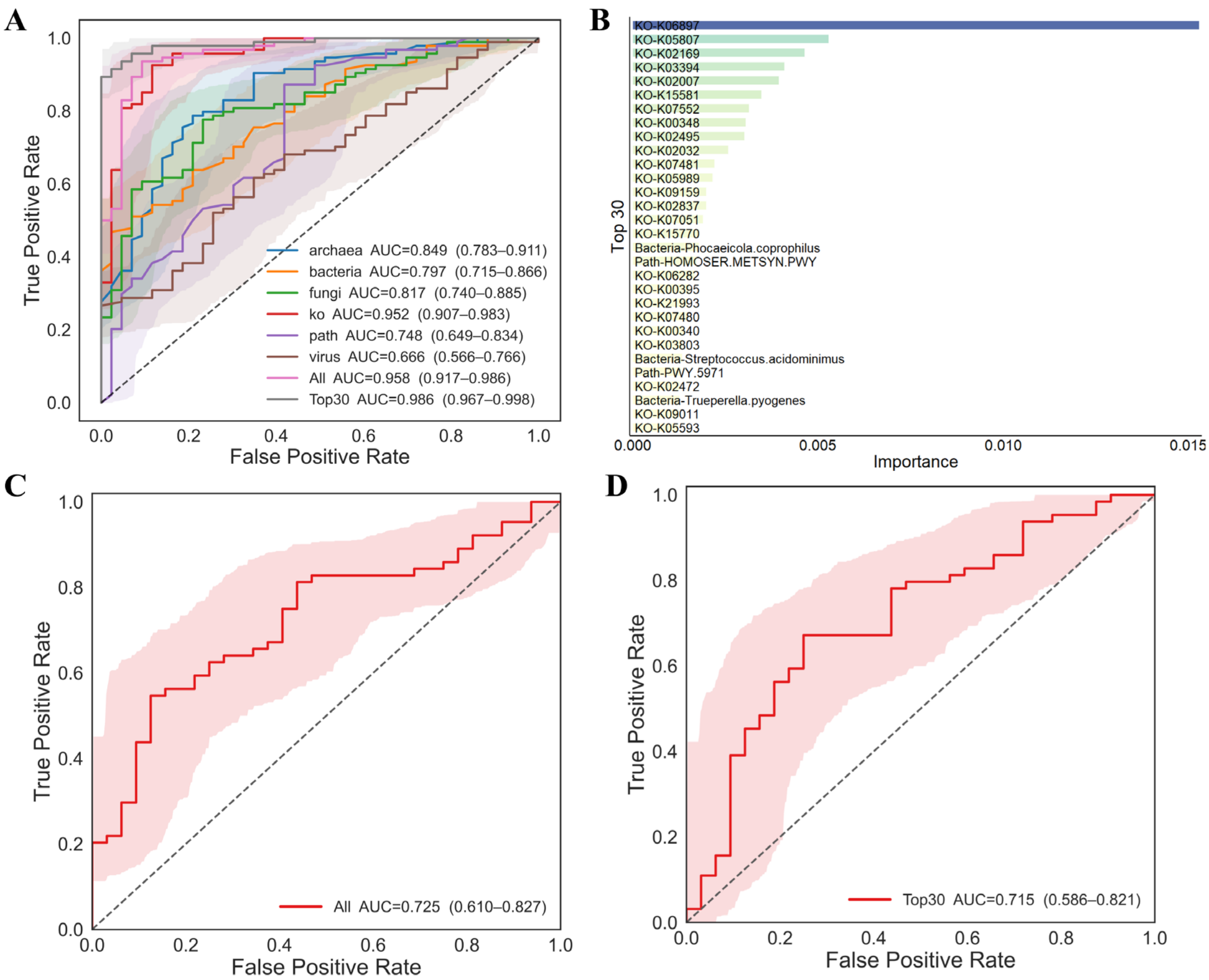

Figure S9. Female-specific random forest models for diagnosing RRMS. (A) ROC curves of random forest models using different microbial features within the test dataset. (B) Importance scores for the top 30 biomarkers identified in the RRMS classification model trained on all microbial features. ROC curves of female-specific RRMS diagnostic model using all microbial features (C) and top 30 features (D) on external validation datasets.

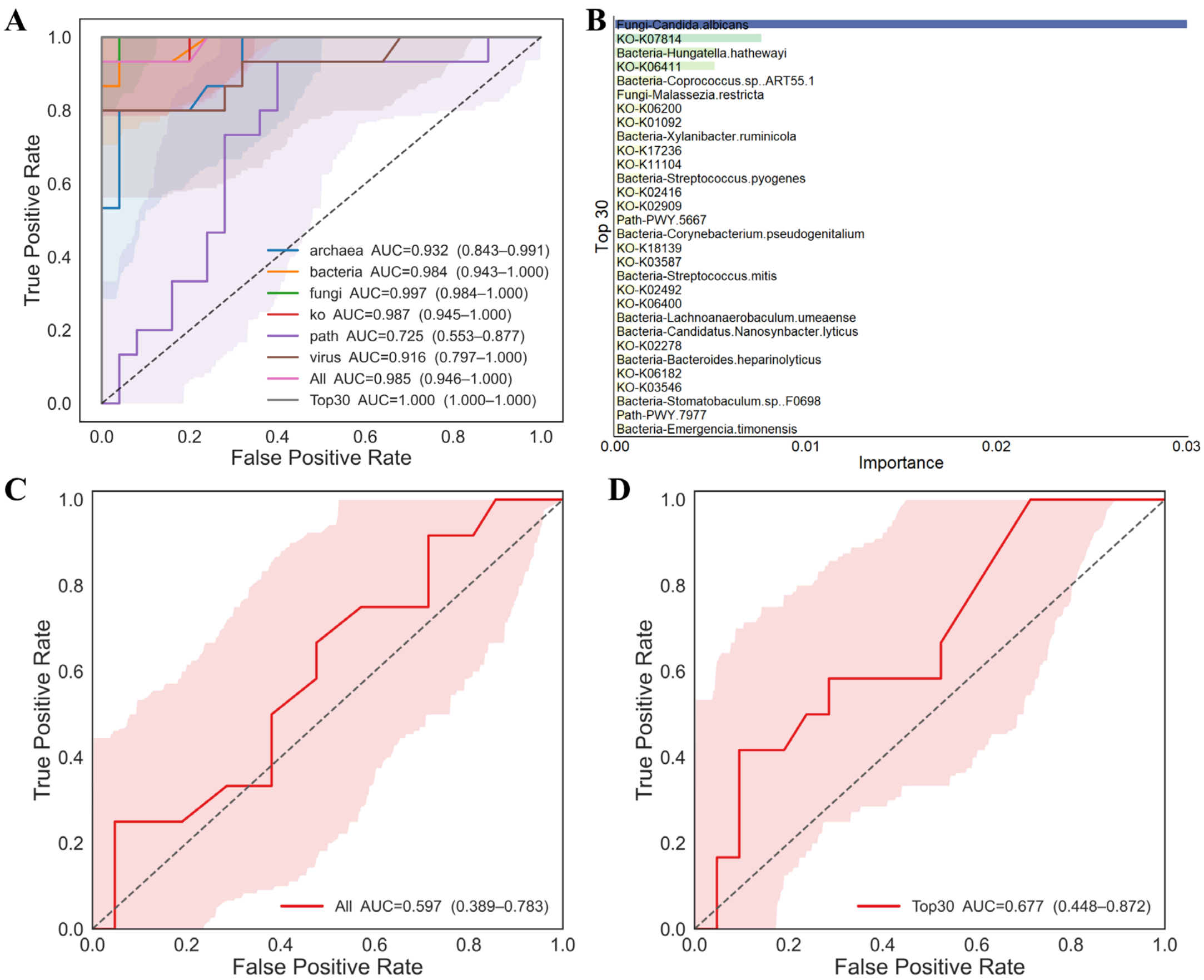

Figure S10. Male-specific random forest models for diagnosing PMS. (A) ROC curves of random forest models using different microbial features within the test dataset. (B) Importance scores for the top 30 biomarkers identified in the PMS classification model trained on all microbial features. ROC curves of male-specific PMS diagnostic model using all microbial features (C) and top 30 features (D) on external validation datasets.

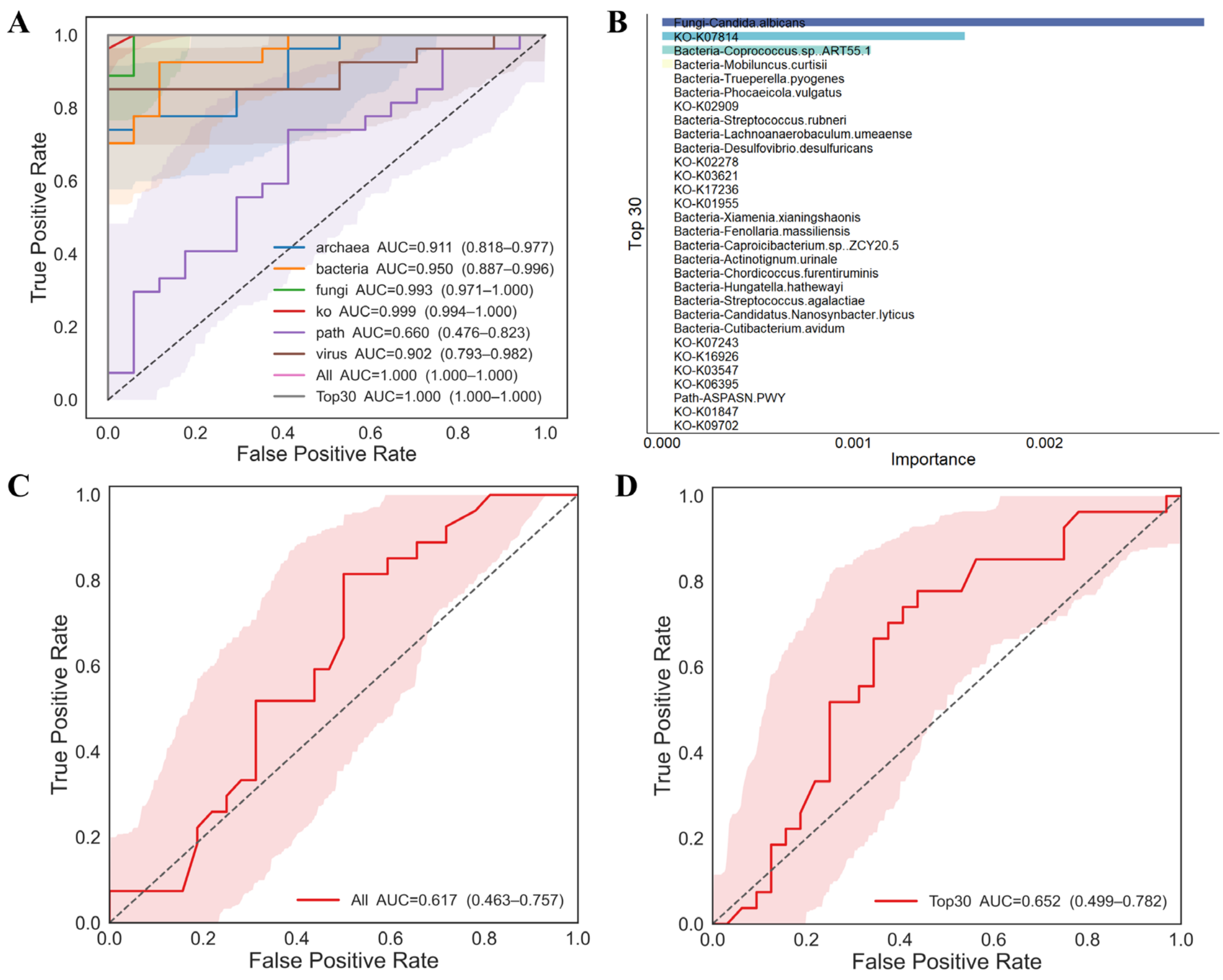

Figure S11. Female-specific random forest models for diagnosing PMS. (A) ROC curves of random forest models using different microbial features within the test dataset. (B) Importance scores for the top 30 biomarkers identified in the PMS classification model trained on all microbial features. ROC curves of female-specific PMS diagnostic model using all microbial features (C) and top 30 features (D) on external validation datasets.

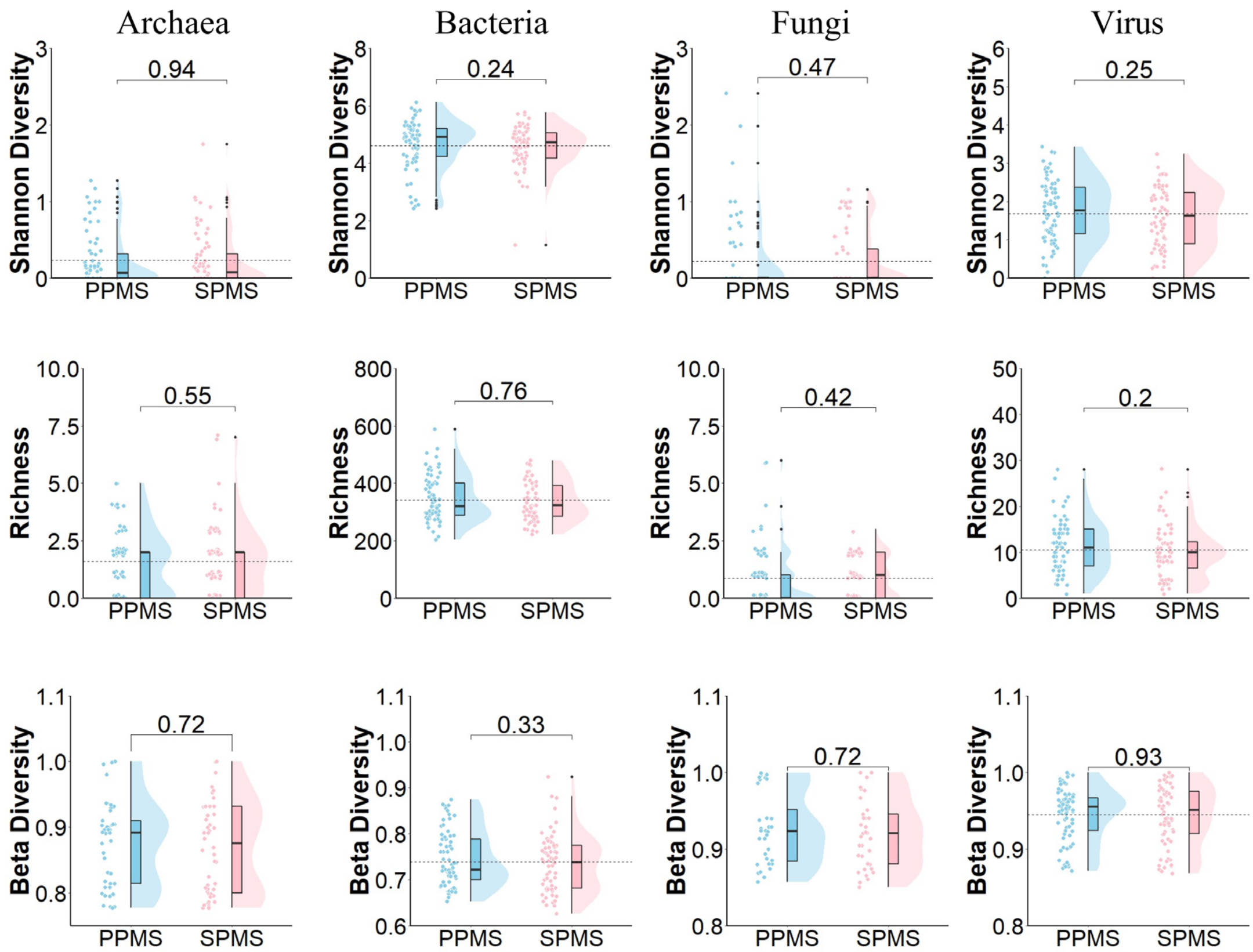

Figure S12. Shannon diversity, Richness, and Beta diversity for the multikingdom microbiome in patients with PPMS (blue, n = 71) versus SPMS (pink, n = 68), assessed by FDR-corrected Wilcoxon rank-sum test.

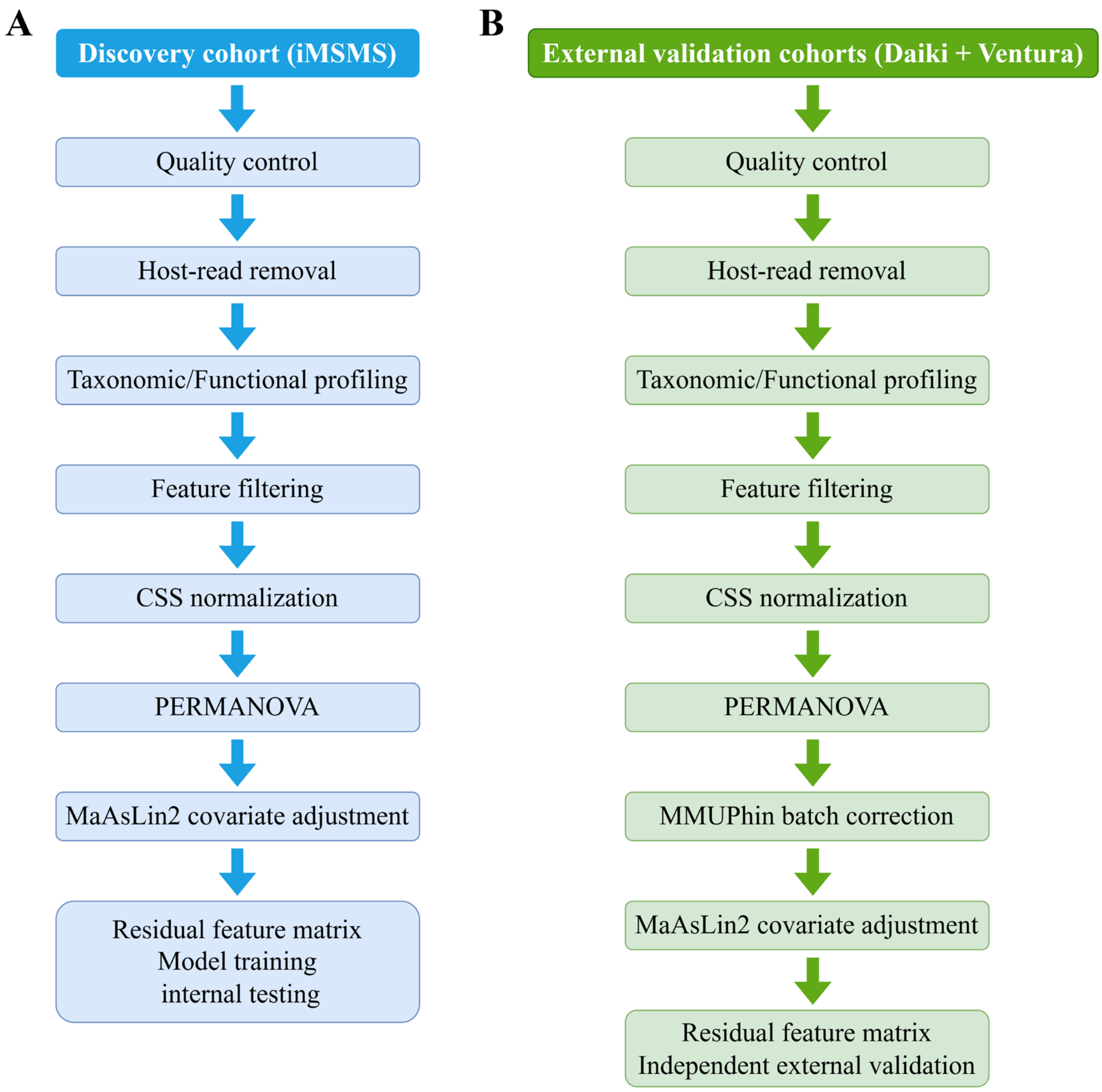

Figure S13. Schematic overview of data preprocessing framework for discovery (A) and external validation cohorts (B).
