# Supplementary material for: Multikingdom microbiome-based machine learning enables multiple sclerosis diagnosis
Source: NPJ Biofilms Microbiomes. 2026 May 30;12:166. doi: 10.1038/s41522-026-01030-7 (PMC13519030; doi:10.1038/s41522-026-01030-7)
Supplement: Supplementary file 2 [file 41522_2026_1030_MOESM4_ESM.docx]

**Table S4. Characteristics of subjects included in this metagenomic study**

| First author | PMID | Group | n | Sex | Age  (ave ± SD) | BMI  (ave ± SD) | Country |
| --- | --- | --- | --- | --- | --- | --- | --- |
| iMSMS | 36113426 | HC | 576 | Female,  34.9% | 50.6 ± 12.8 | 26.9 ± 5.2 | USA,  Argentina,  United Kingdom,  Spanish |
|  |  | RRMS | 437 | Female,  71.4% | 45.8 ± 11.4 | 25.4 ± 5.4 |  |
|  |  | PMS | 139 | Female,  63.3% | 58.6 ± 8.8 | 25.3 ± 4.8 |  |
| Daiki | 32839304,  39772393 | HC | 29 | Female,  58.6% | 38.6 ± 2.3 | 21.4 ± 0.4 | Japan |
|  |  | RRMS | 62 | Female,  74.2% | 39.0 ± 1.0 | 22.2 ± 0.3 |  |
|  |  | SPMS | 15 | Female,  60.0% | 43.3 ± 2.6 | 21.3 ± 0.7 |  |
| Ventura | 31705027 | HC | 24 | Female,  62.5% | 33.5 ± 10.1 | NA | USA |
|  |  | RRMS | 24 | Female,  75.0% | 36.9 ± 12.0 | NA |  |
